# Determinants of COVID-19 vaccine uptake: evidence from a vulnerable global South setting

**DOI:** 10.1186/s13104-024-06736-5

**Published:** 2024-03-29

**Authors:** John Oti Amoah, Susanna Aba Abraham, Charles Atanga Adongo, Deogratias Kaheeru Sekimpi, David Cudjoe Adukpo, Dorcas Obiri-Yeboah, David Teye Doku

**Affiliations:** 1 Centre for Gender Research, Advocacy and Documentation, College of Humanities and Legal Studies, PMB, University of Cape Coast, Cape Coast, Ghana; 2 Department of Adult Health, School of Nursing and Midwifery, College of Health and Allied Sciences, University of Cape Coast, Cape Coast, Ghana; 3 Department of Tourism and Hospitality Management, College of Humanities and Legal Studies, University of Cape Coast, Cape Coast, Ghana; 4 School of Hospitality and Tourism, University of Johannesburg, Johannesburg, South Africa; 5 Uganda National Association of Community and Occupational Health, P. O. Box 12590, Kampala, Uganda; 6 Department of Physics, School of Physical Sciences, College of Agriculture and Natural Sciences, University of Cape Coast, Cape Coast, Ghana; 7 Microbiology and Immunology Department, School of Medical Sciences, College of Health and Allied Sciences, University of Cape Coast, Cape Coast, Ghana; 8 Clinical Microbiology/Public Health Unit, Cape Coast Teaching Hospital, Cape Coast, Ghana; 9 Department of Population and Health, College of Humanities and Legal Studies, University of Cape Coast, Cape Coast, Ghana; 10 Directorate of Research Innovation and Consultancy, University of Cape Coast, Cape Coast, Ghana

**Keywords:** COVID-19 vaccination, Hesitancy, Ghana, Decision-making factors, Public health

## Abstract

**Objective:**

Studies are paying increasing attention to complex social determinants in explaining the variation in the rates COVID-19 vaccine uptake. This study examines the influence of various individual, contextual, and vaccine-related factors on COVID-19 vaccine uptake behaviour in a resource-scarce and vulnerable setting using a quantitative research approach. Using a multi-staged cluster sampling approach, 408 individuals from 204 households in Cape Coast, Ghana’s tourism hub, were surveyed. Probit and logistic regression models were estimated to test the vaccine-related factors.

**Results:**

A significant difference is observed between wait time and vaccination status (χ^2^ = 21.17; p = 0.000). Moreover, age and religion, as controlled variables, equally played significant roles in influencing the adoption of the vaccine. Other factors encompass the perceived risk of contracting COVID-19, the perceived benefits of the vaccine in relation to its side effects, and the level of trust individuals have in the concern of vaccine producers for their health. These findings call for targeted campaigns by the Ministry of Health, health facilities and other institutions promoting the COVID-19 vaccine. These actors should emphasize the benefits of vaccination, prioritize trust-building initiatives, and provide clear guidance on vaccination schedules, and manage waiting time.

**Supplementary Information:**

The online version contains supplementary material available at 10.1186/s13104-024-06736-5.

## Introduction

Ghana is among the 20 countries most impacted in the WHO Africa region [1] and confirmed over 500 new cases and approximately fifteen deaths daily. Through the COVAX initiative, Ghana secured and delivered COVID-19 vaccines to its citizens [2, 3]. The WHO has cited vaccine hesitancy among the top 10 health threats globally [4].

Vaccine hesitancy is a multi-faceted phenomenon caused by an array of issues. In the context of the COVID-19 vaccine, the historic speed at which the vaccine was developed contributes to hesitancy among many populations [5, 6]. To effectively combat vaccine preventable diseases including COVID-19, herd immunity is essential; hence, a high rate of vaccinations in every country is critical. Currently, several vaccines against COVID-19 have been developed (e.g., Pfizer-Biontech, Sputnik V, Moderna, Sinopharm), however, voluntary acceptance and uptake of the vaccines remain a challenge. Despite the WHO declaring COVID-19 no longer a pandemic, forecasts indicate that its risks will continue to persist. Studies have employed various models to explain vaccine uptake behaviour [4]. One strand of the literature concludes that vaccination decisions are underpinned by vaccine features and group and individual contexts [5, 6]. Overall, vaccine hesitancy is a complex phenomenon, particularly in low resource settings [7]. Schwartz [8] explains that rational individuals make health decisions to maximize their self-interest by evaluating the risks and benefits of certain behaviours based on the information they acquire on the issue under consideration. However, there is little literature on how these contextual aspects influence vaccination decision-making and uptake in Ghana.

This study examines the influence of various individual, contextual, and vaccine-related factors on COVID-19 vaccine uptake in a resource-limited and vulnerable setting using a quantitative approach. Vaccine uptake, in this context, is defined as the complete adoption of the recommended number of doses of vaccines necessary to provide individuals with immunity [4].

## Main text

### Methods

#### Study design and site

We conducted an in-person survey in Cape Coast Metropolis in the Central Region of Ghana. The setting was chosen because it is one of the regions in Ghana which had a high number of COVID-19 cases and related deaths. Out of the target eligible individuals for vaccination, 1,693 530, about 854,206 persons representing about 50% had been fully vaccinated by February 2023.

#### Population and sampling procedure

The study participants were selected using a multi-stage sampling technique. From the two sub-metropolitan areas (Table 1). Then, 204 households within those communities were randomly selected in a serpentine order.

**Table 1:** Overview of Category of Respondents

Category of respondents	Sub-Metro	Zone	Communities	Sample
Households	Cape Coast North	Efutu-Kakomdo-Mempeasem	Anto Esuekyi	100
		Abura-Adisadel-Pedu-Nkanfoa	Adisadel Zongo	101
	Cape Coast South	Anakyin-Bakaano-Chapel Square	Chapel Square	104
		Gyegyem Instin-Krootown	Ayikoo-Ayikoo	103

#### Data collection

The survey questionnaire was developed for this study (Additional file 1). The questionnaire was structured into three sections: socio-demographics, determinants of COVID-19 vaccine acceptance and hesitancy. The questionnaire was translated into digital version using an Open Data Kit (ODK) platform and the administration of the survey done using android-enabled tablets.

#### Data analysis

The quantitative data were analyzed using STATA. Descriptive statistics were computed to present the background characteristics of the participants. A chi-square was then computed to explore the associations and differences between the potential explanatory variables and the dependent variable, which is the uptake of the COVID-19 vaccine. Subsequently, multivariate probit and logistic regression models were specified and estimated to examine the influence of the multiple significant variables identified from the chi-square analysis on the dependent variable. Both probit and logistic models were estimated as a consistency check on the robustness of the variables found to be significant in explaining the uptake of the COVID vaccine. Post estimation techniques, including the Wald, goodness of fit, and Hosmer–Lemeshow tests, were used to verify the robustness of the regression results. Model fit indices indicated that both models were reasonably specified and fitted. For example, the Hosmer and Lemeshow probability value for the logistic model was greater than 0.05 (χ2 = 13.47, p > 0.05), and the Omnibus test's model coefficient of chi-square value was less than 0.05 (χ2 = 192.06, P = 0.00). These results indicate that a statistically significant influence is exerted by the independent variables on the dependent variable, vaccination.

**Fig. 1:**
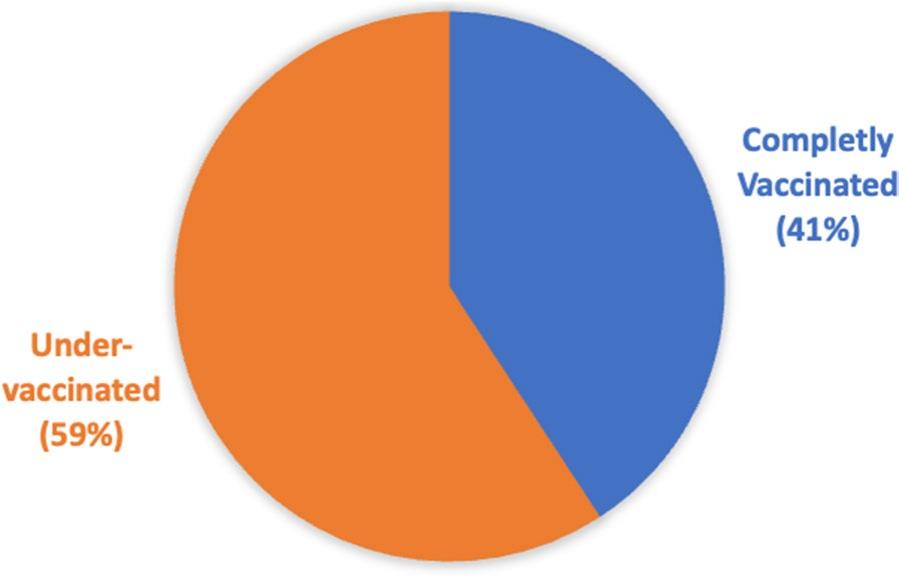
Vaccination status

The dependent variable, uptake, is made up of fully vaccinated (coded as 1) and under-vaccinated (coded as 0). The under-vaccinated group consists of individuals who either refused to take the vaccine despite its availability or those who initiated the vaccination process but did not complete all the required doses. This categorization specifically applies to the AstraZeneca vaccine, which typically requires multiple doses for full immunity. The independent variables include wait time to be vaccinated, perceived benefit of vaccines, source of information of vaccination, and confidence in vaccine safety and efficacy. More than half (59%) of the respondents were under-vaccinated. Of those, about 39% had not been vaccinated and 20% had started but did not complete the vaccination (Fig. 1).

## Results

### Socio-demographic characteristics

Table 2 presents the background characteristics of the study participants. The mean age of the respondents was 42 years, with males accounting for about 65 percent of the responses. Those employed were also the majority (59.7%) and about 70 percent of respondents professed Christianity as their religion. The number of respondents with no formal education (53%) was slightly higher than those with formal education. Of those with formal education, secondary school levers were the majority. The sample consisted of more unmarried participants.

**Table 2:** Socio-demographic Characteristics of Respondents

Variable	Frequency (N)	Percentage
Sex		
Male	144	64.71
Female	264	35.29
Employment status		
Employed	241	59.07
Not employed	167	40.93
Education		
No formal education	217	53.19
Secondary	150	36.76
Tertiary	41	10.05
Religion		
Christianity	285	69.85
Islam	115	28.19
Others (e.g. African Traditional	8	1.96
Marital status		
Unmarried	253	62.01
Married	155	37.99
Age	Mean = 41.72; Minimum = 18 and max = 90; SD = 17.69	

### Bivariate differences in vaccination status by demographic characteristics

The contextual influences included in the regression models were first explored using a chi-square test to ascertain if any variation existed in vaccination uptake status by those characteristics (Table 3). The results revealed that uptake of the COVID vaccine significantly varied by religion (χ^2^ = **5.776;** p = 0.056) and age (χ^2^ = **15.199**; p = 0.010). The data reveals that there is a substantial difference in COVID-19 vaccine uptake between Muslims and Christians, with approximately 10% more Muslims reporting being vaccinated compared to Christians. Additionally, when compared to other religious groupings such as African Traditional religion, there is a significant difference, with about 34% more Muslims reporting vaccination. Generally, none of the age cohorts had 50% of the respondents fully vaccinated, but for those aged 50–59 years (54.24%) because of increased vulnerability and severity should they contract the disease. Under-vaccination was significantly high (72.32%) among respondents aged 20–29.

**Table 3:** Demographic characteristics by vaccination status

	N =	Under-vaccinated	Completely vaccinated	chi2(pr)
Sex				
Female	264	55.89	44.11	3.3930 (0.065)
Male	144	65.28	34.72	
Employment status				
Employed	241	60.00	40.00	0.149(0.699)
Not employed	167	58.08	41.92	
Education				
No formal education	216	61.57	38.43	1.2418(0.537)
Secondary	150	57.33	42.67	
Tertiary	41	53.66	46.34	
Religion				
Christianity	285	61.75	38.25	5.776(0.056)*
Islam	115	51.30	48.70	
Others (e.g. African Traditional	8	85.71	14.29	
Marital status				
Unmarried	253	58.50	41.50	
Married	154	60.39	39.61	0.1418(0.706)
Age				
Below 19 years	24	52.17	47.83	
20–29 years	112	72.32	27.68	15.199 (0.010) *
30–39 years	66	56.06	43.94	
40–49 years	70	62.86	37.14	
50–59 years	59	45.76	54.24	
60 years and above	77	51.95	48.05	
	Mean = 41.72; Minimum = 18 and max = 90; SD = 17.69			
Close family member older than 70				
No	229	58.08	41.92	0.279(0.597)
Yes	178	60.67	39.33	
Living together with close family member older than 70 years				
Yes	339	59.59	40.41	0.117(0.732)
No	68	57.35	42.65	

The significance value has been provide in bold

### Multi-variate regression analysis on the determinants of the COVID-19 vaccine uptake

Table 4 presents both the probit and logistic regression results. Both models revealed five main factors that explained around 22% of the variation in COVID-19 vaccine uptake among the surveyed residents of the Cape Coast Metropolis. These factors are waiting time for vaccination, the belief in vaccine producers' concern for individual health, the perception of vaccination as a means to achieve freedom from restrictions and enhance quality of life, the conviction that the high prevalence of illness is not due to oth of the risk of COVID-19 being greater than the risk of vaccine side effects. To interpret the influence of these factors, we refer to the odds ratio (Table 4).

**Table 4:** Probit and logistic regressions: determinants of uptake of the COVID-19 vaccination among residents of the Cape Coast Metropolis, Ghana

**Determinants **	Model I (Probit Regression)			Model II (logistic Regression)		
	Coef (SE)	P >|z|	[95% Conf. Interval]	Odds (SE)	P >|z|	[95% Conf. Interval]
Gender (ref. male)						
Female	0.1480 (0.152)	0.332	[− 0.151; 0.447]	1.226(0.3089)	0.417	[0.770 2.108]
Religion (ref. others)						
Christianity	0.5421(0.583)	0.352	[− 0.600; 1.684]	3.467(4.80)	0.369	[0.360; 33.381]
Islam	0.939 (0.593)	0.113	[− 0.222; 2.102]	6.542(9.261)	0.185	[0.408; 104.881]
Age	0.005(0.004)	0.162	[− 0.002; 0.013]	1.010 (0.007)	0.151	[0.996; 1.023]
Wait time (ref. Indifferent with the time)						
Less than 30 min	0.423 (0.184)	0.019*	[0.071; 0.796]	2.080 (0.673)	0.024*	[1.102; 3.925]
Maximum 45 min	0.658 (0.297)	0.027*	[0.075; 1.241]	3.013 (1.527)	0.030*	[1.115; 8.139]
An hour and above	0.180 (0.278)	0.518	[− 0.365; 0.725]	1.370 (0.637)	0.498	[-0.365; 0.725]
Trust for pharmaceuticals to make safe vaccines (ref. No)						
Yes	0.077 (0.181)	0.669	[− 0.277; 0.432]	1.204 (0.371)	0.546	[0.657; 2.206]
COVID-19 invented (ref. No)						
Yes	− 0.278(0.155)	0.073	[− 0.583 0.0258]	0.636 (0.169)	0.090	[0.377; 1.073]
Source of information (ref. Others)						
Health care workers	0.303 (0.160)	0.059	[− 0.011; 0.617]	1.658 (0.456)	0.066	[0.967; 2.844]
Vaccine producers interested in your health (ref. No)						
Yes	0.873 (0.206)	0.000*	[0.469; 1.277]	4.109(1.461)	0.000*	[2.046; 8.251]
Benefits- Once vaccinated I will be able to live my life with no restrictions (ref. No)						
Yes	0.513 (0.196)	0.009*	[0.127; 0.898]	2.402(0.813)	0.010*	[1.237; 4.66]
There are no other reasons why so many people are sick (ref. No)						
Yes	-0.8977(0.319)	0.005*	[− 1.523; − 0.272]	0.213 (0.125)	0.009*	[0.067; 0.6758]
My risk of getting sick with COVID‐19 is bigger than the risk of side effects from the vaccine (ref. No)						
Yes	0.444(0.179)	0.013*	[0.091; 0.796]	2.0577 (0.624)	0.017*	[1.135; 3.730]
It is impossible to get COVID‐19 or any other disease from the vaccine itself or its components (ref. No)						
Yes	− 0.046 (0.195)	0.813	[− 0.429; 0.3375]	0.942 (0.313)	0.859	[0.491; 1.808]
Constant	− 2.702(0.650)	0.000	[− 3.978; − 1.427]	0.007(0.012)	0.003	.[ 0.000; 0.1822]

Probit model: Pseudo R.^2^ = 0.2147; Wald chi2(15) = 106.16; Prob > chi2 = 0.000

Logistic model: Pseudo R.^2^ = 0.2147; Wald chi2(15) = 90.02, Prob > chi2 = 0.000

The maximum waiting time for vaccination uptake also had a significant impact on the adoption of the COVID-19 vaccine. Individuals who were willing to wait less than 30 min (OR = 2.080; p = 0.024) or up to 45 min (OR = 3.013; p = 0.030) were more likely to get vaccinated compared to those who were indifferent about the waiting time. However, individuals who indicated a willingness to wait for at least an hour showed a positive influence on vaccine uptake, but the difference was not statistically significant compared to those who were uncertain about the time they would be willing to wait in a queue for vaccination.

Those who perceived vaccine producers were interested in one’s health had a higher chance of completing their vaccination against COVID (ORR = 4.109; p = 0.000) than those who felt that vaccine producers were not concerned about their health. The belief that there are no other reasons why so many people are sick had a significant inverse influence on the uptake (ORR = 0.213; p = **0.009)**, which is an indication that when people perceive that there are varied root causes of an ongoing pandemic and not necessarily the vaccine-preventable disease, it undermines vaccination compliance.

However, when individuals perceive that the risk of contracting COVID-19 is significantly greater than the risk of experiencing side effects from the vaccine, it significantly increases the likelihood of vaccine uptake by 2.057 times (p = 0.015). Such an impression influenced uptake more than those without the impression. This reinforces the fact when perceived vulnerability to the vaccine-preventable disease outweighs the risk of side effects from the vaccine, people are likely to vaccinate against the disease. The belief that being vaccinated would lead to freedom from restrictions and containment measures to control the spread of the virus significantly motivated vaccine uptake, increasing the likelihood by 2.402 times (p = 0.010). In contrast, individuals who held the perception that getting vaccinated against COVID-19 would not enable them to live their lives without restrictions had a decreased likelihood of receiving the vaccine.

## Discussion

Using actual vaccination-behaviour data, this study examined the influence of various individual, contextual, and vaccine-related factors on COVID-19 vaccine uptake in a resource-limited and vulnerable setting. Contrasting findings are observed between the multivariate and bivariate analyses on the influence of contextual factors such as sex, religion and age on vaccine uptake could be attributed to the complex interplay between multiple factors which may mask or attenuate the effects of individual socio-demographic factors. A higher proportion of under-vaccination is observed among Christians and individuals who reported practicing other religions, such as African Traditional religion, when compared to Muslims. De Figueiredo et al. [9] in their study in 149 countries between 2015 and 2019**.** Observed that in instances of lower likelihood of uptake of vaccines, it is religious groups that constitute the minority in the population that showed a significant link with uptake. The refusal to be vaccinated on religious grounds is in line with previous studies, which have established that some people avoided vaccination based on religious incompatibility such as linking vaccines with Satanism and “punishment from God” [10, 11].

Respondents over 50 years were more likely to accept than the younger respondents (i.e., respondents below 30 years). This finding on the age dynamics is consistent with recent studies in Northeast Ethiopia and Western India [10, 12]. In contrast, Samo et al., [2] in a cross-section study conducted in Pakistan found that vaccine refusal was higher in people aged over 30 who live in rural areas. This observation could be attributed to differences in geographical space and exposure to different sources of COVID-19 information among younger people.

The motivation for full vaccination was driven by individuals’ perception of vulnerability to COVID-19 and the perceived benefits of the vaccine. This suggests that a cost–benefit analysis based on protection motivation influenced their decision, where the perceived protection offered by the vaccine outweighed any perceived or real side effects as barriers to uptake [4]. As regards perceived vulnerability and uptake, individuals who believed that their risk of contracting COVID-19 was greater than the risk of vaccine side effects had lower rates of under-vaccination. This finding is consistent with studies in other countries, which found that people who feel threatened are more likely to get vaccinated against COVID-19 [13, 14].

One novelty of this study is that individuals willing to wait shorter periods had lower odds of under-vaccination odds than those who were indifferent or willing to wait longer. This demonstrates greater eagerness or urgency to receive the vaccine compared to those indifferent or willing to wait longer. The odds ratios of 2.080 and 3.013 indicate that individuals willing to wait less than 30 min and up to 45 min, respectively, have significantly higher odds of getting vaccinated compared to those who are more willing to wait longer periods (an hour or more). While contradictory to normative expectations, the insights unpack the complexity surrounding time use in vaccination and re-echo the importance of considering managing vaccination schedules during rollouts.

## Conclusion

Despite ongoing efforts, COVID-19 vaccine sub-optimal vaccination endures. We identified contextual factors such as age and religion, individual and social influences like perceived vulnerability to the disease and vaccine and vaccination-related issues such as the weighing of vaccine benefits against side effects, along with vaccination schedule (wait time), as key drivers of vaccine uptake among households in the Central Region of Ghana. To address these challenges, targeted campaigns by the Ministry of Health, health facilities and other institutions promoting the COVID-19 vaccine should emphasize the benefits of vaccines, prioritize trust-building initiatives, and provide clear guidance on vaccination schedules, and manage waiting time.

## Limitations

Our study relied on a sample drawn from a single municipality; therefore, the findings need to be interpreted in this context. Thus, the findings can only be generalised to similar contexts and populations.

### Supplementary Information

**Additional file 1.** Survey instrument: determinants of covid-19 vaccine uptake: evidence from a vulnerable global south setting.

## Data Availability

The data that support the findings in this study are available from the corresponding author upon reasonable request.

## Abbreviation

PHSM: Public Health and Social Measures
