# Supplementary material for: Determinants of COVID-19 vaccine uptake: evidence from a vulnerable global South setting
Source: BMC Res Notes. 2024 Mar 29;17:94. doi: 10.1186/s13104-024-06736-5 (PMC10979621; doi:10.1186/s13104-024-06736-5)
Supplement: Supplementary file 1 — Additional file 1. Survey instrument: determinants of covid-19 vaccine uptake: evidence from a vulnerable global south setting. [file 13104_2024_6736_MOESM1_ESM.pdf]

ADDITIONAL FILE 1: SURVEY INSTRUMENT: DETERMINANTS OF COVID-19 VACCINE UPTAKE: EVIDENCE FROM A VULNERABLE GLOBAL SOUTH SETTING

SURVEY INSTRUMENT: QUESTIONNAIRE FOR HOUSEHOLD MEMBERS ABOVE 18 YEARS

Introductory tasks:

1) **Welcome participants**
2) **Introduce yourself and Project**
3) **Explain research and assure confidentiality/anonymity where applicable**
4) **Confirm consent for participation and tape recording (sign consent forms)**

Introduction

Good morning/afternoon, my name is ________________ and I'm a researcher from CEGRAD, UCC conducting today's research on COVID-19 vaccination decision making processes. Access to information on vaccination varies between urban and rural settings and so does cultural believes and practices. This study seeks to explore *determinants of COVID-19 vaccination-related decisions making by individuals* in Cape Coast Metropolitan in the Central Region of Ghana. It is important to examine this issue of COVID-19 vaccine decision making process across the sociocultural divide to inform tailored interventions.

I want to thank you greatly for your interest and willingness to participate. We are anticipating that the discussion will last for about one hour or 45 minutes.

Confidentiality/Anonymity:

All information that I collect during this discussion will be confidential. If we quote anything you say in a subsequent report, your name will not be used but your position may be used. You will only be identified as a community member in this community. We will record the session but all responses will appear anonymously.

Risks and Discomforts:

There is a risk that you may discuss something that a colleague might disagree with. Therefore, you may not have to answer any questions or participate if you are not comfortable. You may leave this discussion at any time you want to.

Consent:

As said earlier, your name will not be identified so you will remain anonymous. I have consent forms that state everything that I have just explained. Although I will ask you to sign/thumbprint, these consent forms will be kept in a secure place and not be used to identify you. Does anyone have any questions concerning this discussion or your rights during this research? **Have participants sign consent forms.**

SECTION A: PERSONAL INFORMATION

- Age (years)
- Sex

- Educational level
- Employment status
- Number of family members
- Marital status
- Religion

1. Have you got any close family members (father/mother/grandfather/grandmother) older than 70 years?

- o Yes, living together
- o Yes, not living together
- o No

2. You would define your family income as…

- o Lower than average
- o On average
- o Higher than average

3. Do you have any of the following conditions? [select all that apply]

- o Cancer
- o Immunocompromised state due to therapy or disease
- o Obesity
- o Diabetes (type 1 or 2)
- o Cardiovascular disease
- o Pulmonary disease
- o Rheumatological condition

SECTION B: PERSONAL EXPERIENCES

4. If you have refused a vaccine in the past that was recommended to you by a healthcare worker - what was/were the reason(s)? [check all reasons that applied to that situation]

- o I never refused a vaccine recommended by a healthcare worker
- o Did not think it was needed
- o Did not have enough information on the vaccine
- o Did not think the vaccine was effective
- o Did not think the vaccine was safe
- o I was concerned about side effects
- o I had a bad experience with a previous vaccination
- o Did not know where to get vaccination
- o Other logistic problems

5. Have you had COVID-19?

- o I had suspected symptoms but I didn't verify with a doctor and/or specific exams
- o No
- o Yes with no symptoms
- o Yes with mild symptoms
- o Yes with severe symptoms

SECTION C: FACTORS ASSOCIATED WITH VACCINE HESITANCY

Vaccination likelihood

6. A COVID-19 vaccine has already been approved. If you were offered to get the vaccine in the next weeks at no cost for you- how likely are you to take it?
   - o Very likely
   - o Somewhat likely
   - o I am not sure
   - o Somewhat unlikely
   - o Very unlikely
   - o I would not take it within the next two months but I might reconsider it in the future

7. What would be important for you to know to make you more confident in the COVID-19 vaccine? [Select up to 3 options]
   - o The fast production of the vaccine did not compromise its safety
   - o Agencies approving the vaccines are following strict rules
   - o My risk of getting sick with COVID-19 is bigger than the risk of side effects from the vaccine
   - o The vaccine cannot cause any immediate or long term injury
   - o It is impossible to get COVID-19 or any other disease from the vaccine itself or its components
   - o The vaccine works in protecting me from COVID-19
   - o The vaccine works in stopping the transmission of COVID-19 from one person to another
   - o Health agencies and WHO recommend the vaccine and agree it is safe
   - o I do not need any other information
   - o Other - please specify__________________

8. What else would be important for you to know to make you more likely to take the COVID-19 vaccine? [Select up to 3 options]
   - o Once vaccinated I will be able to live my life with no restrictions
   - o Those with concerns about the vaccine have opportunities to share their opinions with the public
   - o Pharmaceutical companies will not make large profits from the vaccine
   - o Everybody will have equal access to the vaccine regardless of income or race
   - o I will be free to choose if I get the vaccine or not with no consequences
   - o There are no other reasons why so many people are sick (i.e. 5G technology or other unknown reasons
9. What is the maximum amount of time you would be able or willing to spend to get a vaccine for yourself?
   - o Less than 30 minutes
   - o Maximum 45 minutes
   - o Maximum 1 hour
   - o Between 1 hour and 2 hours
   - o Indifferent with the time

Religion/culture/gender

10. Does your religion recommend against (a certain) COVID-19 vaccine?
    - o Yes
    - o No

11. If so, which vaccines?
    - AstraZeneca
    - Sputnik-V
    - Moderna
    - Pfizer/ BioNTech
    - Johnson & Johnson
    - All
12. What is the reason for the recommendation?
    -

13. Would you refuse a COVID-19 vaccine if the vaccinator was male or female?

    - Yes
    - No
14. Would you refuse a COVID-19 vaccine if the vaccinator was from a different religion than yourself?
    - Yes
    - No
15. Would you refuse a COVID-19 vaccine if the vaccinator was from a different ethnic group than yourself?
    - Yes
    - No
16. Would you refuse a COVID-19 vaccine if the vaccinator appears younger than yourself?
    - Yes
    - No

SECTION D: EXPLORE KNOWLEDGE AND ATTITUDE TOWARDS COVID-19 VACCINATION

17. Who do you trust the most for COVID-19 vaccination information?
    - Health care workers
    - Religious leaders
    - Traditional Leaders
    - Politicians
    - Traditional Media
    - Social media
18. Who do you trust the least for COVID-19 vaccination information?
    - Health care workers
    - Religious leaders
    - Traditional Leaders
    - Politicians
    - Traditional Media
    - Social media
19. Have reports you read or heard in the media/ on social media made you re-consider the choice to get vaccinated?
    - Yes
    - No
20. Do you trust pharmaceutical companies to provide safe and effective COVID-19 vaccines?
    - Yes
    - No
21. Do you believe vaccine producers are interested in your health?
    - Yes?

- No?

SECTION E: MYTHS RELATING TO COVID-19 VACCINATION

22. Do you believe the reports in the media/ social media that claim that the COVID-19 vaccine can cause [select all that apply]
    - Infertility in men
    - Infertility in women
    - Cancer
    - Death
    - Other - please specify___
23. Did you ever have the impression that the government did not opt for the best vaccine on the market?
    - Yes
    - No
24. Do you believe the reports in the media/ social media that COVID-19 was invented by scientists?
    - Yes
    - No
25. Do you believe the reports in the media/ social media that COVID-19 vaccines were not rigorously tested?
    - Yes
    - No

THANK YOU
